# A computed tomography (CT) study of the eyeball position and estimation models for craniofacial identification

**DOI:** 10.1007/s00414-026-03768-3

**Published:** 2026-04-10

**Authors:** Robert A. R. Humphrey, Te Wai Pounamu T. Hona, Carl N. Stephan

**Affiliations:** Laboratory for Human Craniofacial and Skeletal Identification (HuCS-ID Lab), School of Biomedical Sciences, The University of Queensland, Brisbane, 4072 Australia

**Keywords:** Craniofacial identification, Craniofacial superimposition, Video superimposition, Facial approximation, Facial reconstruction, Eye projection, Globe projection, Orbit

## Abstract

Correctly setting the eyes within the orbits is an important first step in craniofacial identification. As several estimators often exist for each eyeball attribute, clarification of their accuracies in out-of-group samples is critical. In this study, we use CT scans from 58 individuals to assess standard errors (SEE) for published estimators, cross-validate methods and examine the linear regression utility of orbit volume for estimating eyeball protrusion. Our results confirm the eyeball to be positioned closer to the superior and lateral orbital walls (2.5 & 5.6 mm, respectively). Horizontal placement was best estimated by the pupil center at 42.4% of the orbit width and medial to the lateral orbital margin (SEE = 1.6 mm). Anterior eyeball position was best set by the grand mean exophthalmos (16.2 mm) measured in living subjects (SEE = 2.9 mm). Vertical eyeball height could be estimated, with equivalent error, by either: (a) setting the pupil center 22.9 mm inferior to the superior orbital margin (SEE = 1.7 mm); or (b) inferior to the superior orbital margin by 44.1% of the orbit height (SEE = 1.6 mm). Orbit volume weakly correlated with anterior eyeball protrusion (*r* = 0.33), but the relationship was not statistically significant (*p* = 0.14). The correlation direction contradicted Gerasimov’s reports (positive, not negative). Linear regression using the volume of an elliptical cone, calculated from three orbit-associated measurements, reduced protrusion SEE to 2.4 mm. Near equivalent accuracy by mean exophthalmos and its far greater simplicity, however, awards the mean value more practicality for casework.

**Supplementary Information:**

The online version contains supplementary material available at 10.1007/s00414-026-03768-3.

## Highlights

Eyeball estimators were assessed by CT and standard errors of the estimate (SEE).

For globe protrusion, mean exophthalmos (16.2 mm) produced the lowest SEE (2.9 mm).

The globe was more superior and lateral in the orbit (2.5 & 5.6 mm, respectively).

Orbit volume weakly correlated with globe protrusion, r = 0.33 (p > 0.05).

A 3D elliptical cone, as a proxy to orbit volume, slightly decreased SEE (2.4 mm).

**Supplementary Information:**

The online version contains supplementary material available at 10.1007/s00414-026-03768-3.

## Introduction

An estimate of the eyeball’s position is pivotal when undertaking craniofacial identification [1]. Craniofacial identification methods include facial approximation and craniofacial superimposition, which are used in the most challenging of skeletal cases when identifications based on other biological methods such as DNA, dental or medical radiographs are not possible [2, 3]. Facial approximation refers to the estimation of a face from a dry skull, with the aim to trigger a facial recognition when the estimated face is advertised in the media [4–6]. Craniofacial superimposition refers to the photographic overlay of a face photograph, taken during life of an individual, with a photograph of a skull, to assess an identity [7, 8]. In both methods, accurate facial feature estimation using scientifically formulated models is preferred [5, 9].

While correct estimation of all major facial features is important, the eyes hold key roles in both methods. For example, the eyes play a primary role for facial recognition [10–12], which earmarks this region as a prime area of concern for facial approximation. For craniofacial superimpositions using frontal view photographs, correct z- and x-axis positioning of the globe on a cartesian coordinate system and within the orbit, is important for establishing interpupillary distance and level of the eyes relative to other facial features. For profile face views in craniofacial superimposition, y-axis placement is critical for establishing correct projection of the corneal apex from the lateral orbital wall and, subsequently, if the skull/face combination represents a plausible anatomical pair.

In the frontal view, the cone-shaped bony eye socket and its four orbital margins provide the physical limits to the eyeball positioning along x- and z- coordinate axes. However, the diameter of the eyeball is considerably smaller than that of the orbital rim, such that there is roughly 10–12 mm of free space on either side of the eyeball within the orbit [13, 14]. This intermediate zone between the eyeball and the hard bony wall is filled mostly by fat [13–15] and provides potential variety between individuals as to where the eyeball is exactly positioned. Clearly, methods that enable positioning more precisely than just the raw limits provided by the bony walls of the orbit, would be useful.

In the literature, some progress has already been made on refining the anatomical details of exact eyeball position relative to the skull. Since 2002, mean exophthalmos data measured in living subjects [16–21] has been proposed for establishing y-axis projection from the deepest part of the lateral orbital wall [22]. Longstanding anatomical observations [23–25] that the eyeballs are positioned closer to the superolateral walls than the medioinferior walls, have also been quantitatively demonstrated [13–15, 22, 26]. Cross-validation using advanced medical imaging techniques like CT (see e.g. [26–28]), has been initiated and these studies have resulted in further refinements. For example, percentages of pupil position relative-to-orbit dimensions have shown promise over and above mean linear displacements for estimations [26]. Orbit depth measurements taken from MRIs [29] have also been used to evaluate the longstanding speculation that globe projection covaries with orbit size/volume [30, 31]; however, the derived estimation models have not been subject to cross-validation tests using independent samples. A lack of such out-of-group testing applies to much of the research described above and this requires attention to determine if the estimation models are reliable and deliver what they claim to achieve outside of their initial training samples.

Of course, present-day medical imaging offers the substantial advantage of visualizing the skull and soft tissues together on the same image, as useful for taking measurements in the craniofacial identification context [26–29, 32]. However, neither CT or MRI are silver bullets and some limitations remain [32]. For example, slice thickness and pitch are important resolution considerations as applicable to visualizing and measuring fine anatomical details [32, 33]. Clearly, higher resolutions are preferred over lower ones [32, 33], but not all medical scans used for data analysis in the craniofacial domain have been attained such thin slice thicknesses that provide good resolutions, see e.g. [34]. Also, when subjects assume the supine position to be scanned, their eyeball is liable to move slightly posteriorly under the downward vector of gravity and against its underlying deformable fatty cushion [32, 35, 36]. Despite this last limitation, CT and MRI nevertheless permit 3D rendering of deep cranial structures encased in soft tissues, that cannot be undertaken with other methods or noninvasively and jointly enables the precise calculation of volumes from 3D volume renders. This is a major advantage of CT/MRI that, so far, has not been utilized to its fullest extent. Instead, simple linear measurements taken from CT and MRI are the predominant mode of measurement from these scans in the craniofacial identification research literature concerning the eyes [26–29].

In the other medical literature, orbit volume has also only been used in one eyeball protrusion study. In this study, extrinsic eye muscles were excluded from the volumetric calculations, rendering a measurement termed *effective orbital volume* [37]. This study by Detorakis et al. [37] found a statistically significant relationship between effective orbital volume and the transverse, but not sagittal, protrusion of the eye. Note here that effective orbital volume is not the same as total orbit volume; nevertheless, this study lends support towards exploring the utility of correlations between eyeball protrusion and total orbit volume to derive newer, more precise, estimation models for craniofacial identification following initial speculations by Gerasimov in this domain some 70 or more years ago [30, 31].

This CT study sets out to: (1) cross validate already proposed models to establish vertical, horizontal and anteroposterior location of the eyeball within the orbit; (2) determine if total orbit volume correlates with eyeball protrusion strongly enough to generate useful regression equations that out-perform arithmetic means of globe projection; and (3) determine if total orbit volume can be simplified to an elliptical cone (based on chord length measurements) to improve ease and practical application of regression-based estimation models for the eye protrusion in forensic casework. The methods to be assessed for aim one are as follows: Stephan et al. [14], Stephan [22], Guyomarc’h et al. [26] and Wilkinson and Mautner [29].

## Materials and methods

### CT sample & images

Full-head CT scans were acquired of 58 embalmed decedents (n_1_) representing body donors to the UQ anatomy program and comprising 34 males and 24 females. Participants’ mean age was 86.3 years with a standard deviation of 7.6 years. CTs were acquired using a 16-slice Biograph helical CT (Siemens Global, Germany) using a slice thickness = 0.75 mm, pitch = 0.5 and a reconstruction increment = 0.5 mm. Other CT settings included: 130 kV, 220 mAs, CT dose volume = 60.48 mGy, dose length product = 1620.26 mGy*cm. The final 58 subject sample was arrived at after eight individuals from a total of 66 subjects were excluded due to at least one globe not being fully inflated.

Decedents were specifically chosen for this study, rather than living subjects, since the thin slice thickness settings used to acquire high-clarity images at a field-of-view size that retained the whole head produced ionizing radiation doses that exceeded permissible levels for clinical scans of living subjects. Relevant here is also that the lens of the eye is an especially radiosensitive organ [38]. A second major advantage of using decedents was that all participants were precisely stationary during scan acquisition (no movement due to breathing, heartbeat, blood pulse, blinking, muscle tone), such that the anatomical clarity of the CT scans was very high. Once acquired, CT images were reviewed in OsiriX MD 14.0.1 (Pixmeo, Switzerland), where the heads were reorientated precisely to Frankfurt Horizontal, ready for measurement. Measurements were taken on one side only, with a preference awarded to the left side (*n* = 45) and the right side reserved only for those cases (*n* = 13) where left side proved to be too challenging or unclear.

### Linear measurements

Most of the measurements taken in this study replicate those used in prior work [13, 14, 22, 26, 29], however, there are some newly defined metrics to enable pursuit of the novel estimators explored in this study. In total, 11 cranial landmarks (Supplementary File 1) were used to define 19 linear measurements (Supplementary File 2), plus orbit volume for a total of 20 measurements, that were collected by the first author (RH). Landmarks were placed using both 3D skull surface renderings (decimate resolution of 0.1 and smoothing of 5) and synced 2D orthogonal views (all three standard anatomical planes used for syncing). For examples, see Supplementary File 1.

Linear measurements provided the capacity to put estimation models previously released in the literature [14, 22, 26, 29] to the test. That is, estimation of globe position in the frontal plane using mean displacement of the oculus anterius from the orbit center of 5.4 mm laterally and 2.5 mm superiorly [14]. This translates to a mean distance from the lateral orbital wall of 17.3 ± 1.5 mm and from the superior orbital margin of 22.9 ± 1.6 mm [14]. So too, grand means from exophthalmic data from living subjects [16–21] that establishes the oculus anterius to be 16.2 mm anterior to the deepest part of the lateral orbital wall (*n* = 1,174) [22] were evaluated. Setting the ocular anterius 44.1% under the superior orbital rim relative to overall orbital height dimension, 42.4% medial to the lateral orbital margin and projecting 51.3% of the orbit height dimension in front of the lateral orbital rim, after Guyomarc’h et al. [26] was additionally tested. Lastly, the linear regression equation for globe projection reported by Wilkinson and Mautner [29] was evaluated using newly collected out-of-group data. This method takes orbit depth, from a tangent at the anterior orbital margin to the optic canal, as the independent variable, namely: globe protrusion = 18.3–0.4*orbital depth [29].

### Orbit volume (mm^3^) as an estimator for globe protrusion

**Fig. 1:**
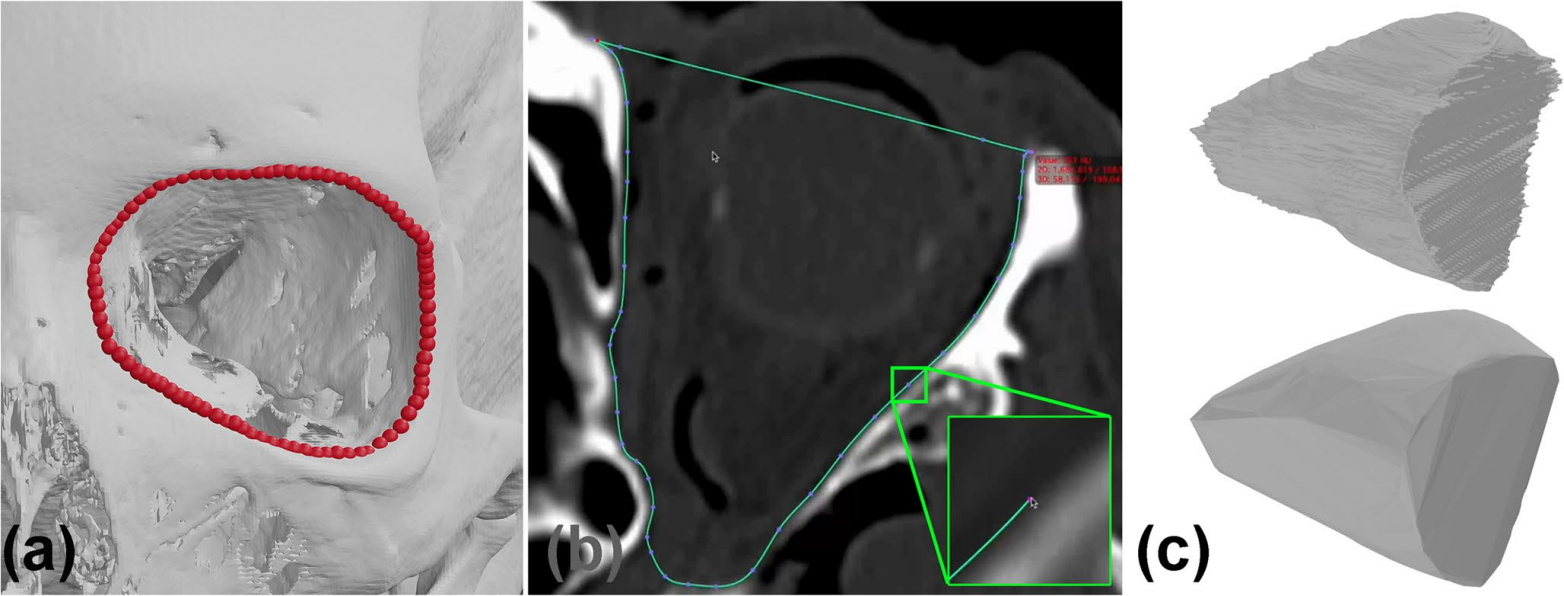
Orbit segmentation in OsiriX MD (Prixmeo, Switzerland): (**a**) final render of an aditus orbitalis with multiple landmarks following protocols of [43] on a left orbit; (**b**) example segmentation (inferior view) on a 2D orthoslice using OsiriX MD’s closed polygon tool (same subject as (**a**)) with an inset to show a zoomed view of the orbit wall segmentation; and (**c**) 3D orbit volume render from the segmented slices from (**b**) in Blender (top image is with iso-counter filter and bottom image is with the Delaunay filter, the latter used for all rendered 3D volumes in this study). For a video example of the segmentation process, see Supplementary File 3

**Fig. 2:**
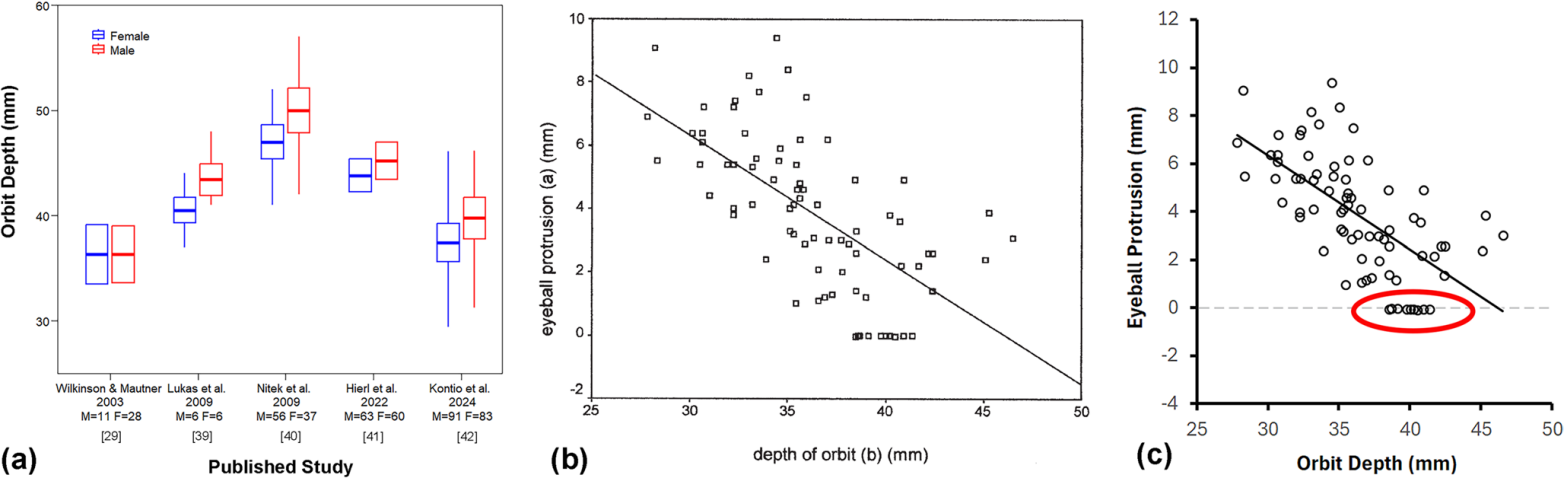
Previously published orbit depth and globe protrusion results: (**a**) mean (horizontal bar), standard deviation (box) and min/max (whiskers) of previously published studies [29, 38–41]; (**b**) original Fig. 3 scatterplot from Wilkinson CM, Mautner SA (2003) Measurement of eyeball protrusion and its application in facial reconstruction. J Forensic Sci 48: 12 − 6. https://doi.org/10.1520/jfs2002053 [29] reproduced with permission from ASTM International; and (**c**) artistic impression of (**b**), created with the help of plotdigitizer.com to show zero line, cluster of points near the zero line margin (red ellipse) and a trimmed regression line. Note that data points in (**c**) are only approximations of (**b**) because they represent artistic impressions, not real values. Also note (**b**) and (**c**) contain more data points than subjects as Wilkinson and Mautner [29] used dual left and right sides from each of 39 subjects in their analysis

The CT scans utilized for measurements in this study enabled the correlation of orbit volume with globe projection to be directly calculated (Fig. 1). This relationship was first implied by Gerasimov some 70 years ago [30, 31] and specifies that orbits with a heavy orbital rims which are long, broad and tall (closed type), carry eyeballs with less substantive projection. Prima fascie, the correlation work of Wilkinson and Mautner [29] using a linear dimension of orbit depth (*r* = −0.646, *p* < 0.001) appears to confirm Gerasimov’s views, however, there are some limitations to the Wilkinson and Mautner study that leave volumetric relationships unclear and requiring further substantiation. For example, in Wilkinson and Mautner’s [29] study: (a) a single linear orbit depth measurement (units = mm) was used as the correlate, not a volume (units = mm^3^); (b) the sample size was small at *n* = 39; (c) correlations were conducted on duplicated samples (both left and right sides of single individuals are included as the data sample) risking inflated strengths of relationships since the data were not independent; and (d) other data oddities appear to be present, e.g., Wilkinson and Mautner’s [29] mean orbit depth is the lowest of any such anatomical investigation so far reported in the literature [39–42] and Wilkinson and Mautner’s [29] own plots show data truncating close to zero and without any negative values, which is strange, i.e., the range is 0–9.4 mm with a cluster on the zero line and no eyeball projecting further anteriorly than the reference tangent located at the front of the orbit (Fig. 2). Additional studies of these items would, thereby, be useful.

Intraorbit volume was calculated from the CT images by first segmenting the inner soft tissues of the orbit from the bony margin at their interface following the protocols of Jansen et al. [43]. In this approach, multiple landmarks were first placed along the orbital rim in OsiriX MD using a 3D volume render view, to later serve as reference points during segmentation on 2D orthoslices (Fig. 1 and Supplementary File 3). To establish the orbit rim, we set the following five landmarks first (supraconchion [sk], orbitale [or], the most lateral aspect of the lateral orbital margin [lLOM], the deepest point on the lateral orbital margin [dLOM], ectochondrion [ec] and Flower’s point [FP]) and then filled intervening segments between the five landmarks with multiple other pseudo-landmarks on the rim, so that all reference landmarks lay side-by-side with minimal intervening space between them and outlining the entire circumference of the aditus orbitalis (Fig. 1 and Supplementary File 3). Excluding the first and last 2D orthoslice, segmentations were conducted on 2D orthoslices separated by two millimeter intervals along the line of z-axis or same direction of the CT table’s movement (Fig. 1). The orbit’s posterior apertures, such as the optic canal, were closed for volume segmentations, by running the segmentation line across the corresponding space (i.e., to discount the canal opening). Similar was also done at the lacrimal bone in reference to the nasolacrimal duct to ensure its exclusion from the orbit volume calculation. Once the orbit had been segmented on all relevant slices, OsiriX MD’s “Compute volume” tool was used to calculate the orbit volume. A video demonstration of an orbit segmentation being undertaken for one individual in OsiriX MD is provided in Supplementary File 3. The “Delaunay” filter in OsiriX MD was used to generate 3D models, which were later exported to Blender 3.3.1 for stacking, subsequent rendering and measurement per methods of Konito et al. [42] (Fig. 1).

### A mathematical proxy to orbit volume, calculated from three linear measurements

**Fig. 3:**
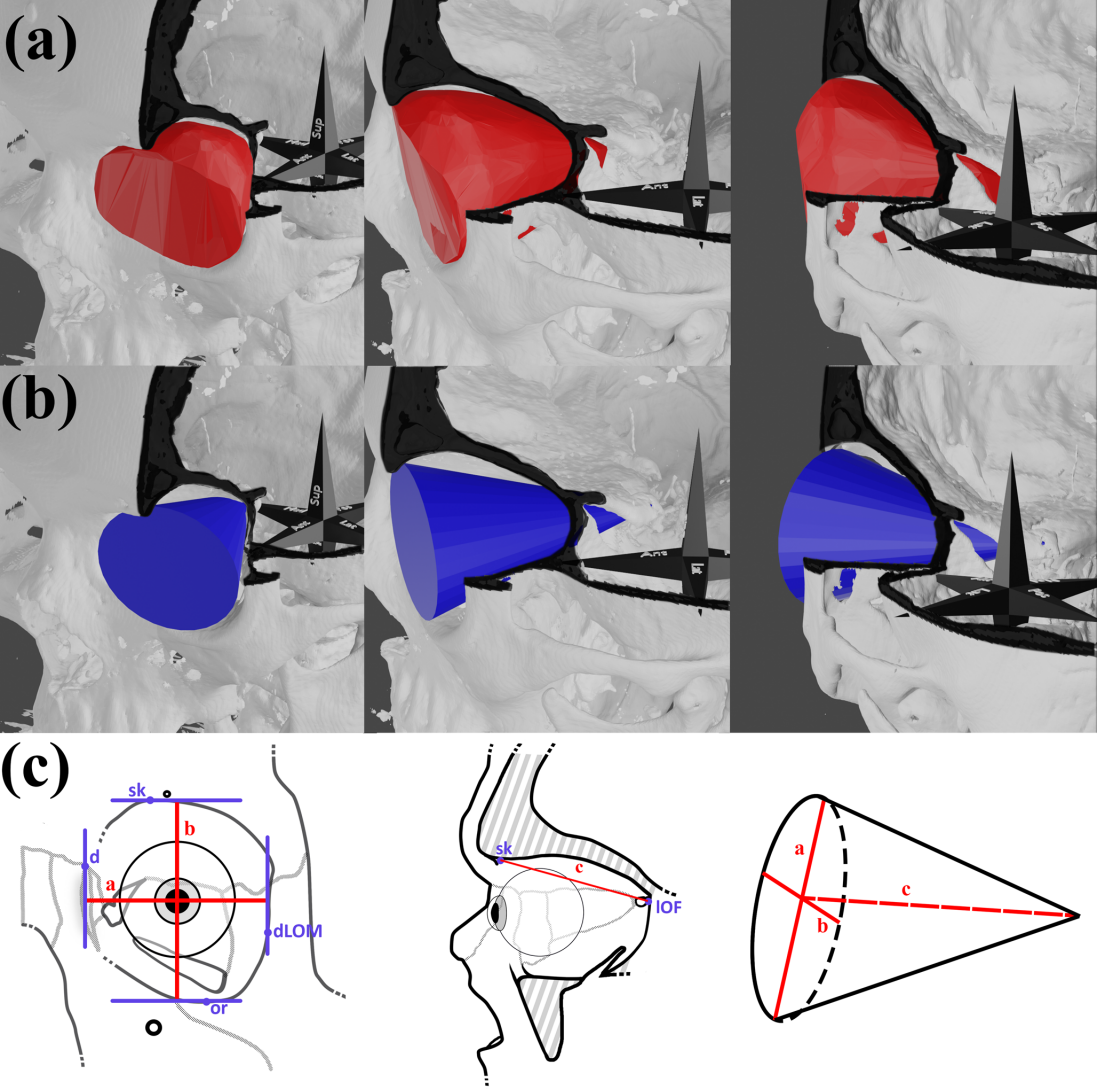
Approximation of the orbit space using an elliptical cone: (**a**) segmented orbit volume in situ with upper quarter left quarter of skull vault removed to provide line of sight to orbit; (**b**) an elliptical cone used to approximate the orbit volume; and (**c**) the three measurements used to mathematically calculate the volume of the 3D elliptical cone: a = orbit height (measurement #5 + #6 in this study), b = orbit width (measurement #8 + #9 in this study), and c = anteroposterior orbit length (sk-lOF)

Since acquiring a volumetric scan in casework can add additional time, expense and require access to a CT scanner, we investigated the additional option to model orbit volume mathematically from three, more easily acquired, linear measurements. We hypothesized this may be useful because the orbit space approximates an elliptical cone─pyramidal in shape with a deep pointed apex and an anteriorly located elliptical base that opens anterolaterally [15, 44] (Fig. 3). The measurements selected for cone height and base analogues were orbit height, orbit width and the cord length distance from supraconchion [sk] to the lateral aspect of the optic foramen [lOF] (Fig. 3). The mathematical equation used was:

1 $$\mathrm{Volume}=\frac13\left(\mathrm\pi\mathrm{*}\frac{\mathrm a}2\mathrm{*}\frac{\mathrm b}2\mathrm{*}\mathrm c\right)$$

Where:

a = orbital height taken at the midsagittal plane of the orbit [sk–or].

b = orbital width taken at the midpoint of a [d–dLOM].

c = supraconchion [sk] to lateral aspect of the optic foramen distance [lOF] (= orbit length).

### Out-of-group validation

CT scans from 18 new individuals (n_2_) who were not used to train any volumetric estimation models mentioned above, were used as an out-of-group sample for cross-validation of any new models derived in this study. This sample comprised 10 males and 8 females with a mean age of 80.8 years and a standard deviation of 11.6 years. These individuals subsequently also provided an opportunity to boost test sample sizes used for already published estimation methods [14, 22, 26, 29], such that data were able to be analyzed for 76 subjects overall (n_1_ + n_2_ = 76). We refer to this pooled total sample as n_3_, that is, n_3_ = n_1_ + n_2_. For completeness, we present the results for all three datasets, including the combined n_3_ dataset, for each of the estimation methods of Stephan et al. [14], Stephan [22], Guyomarc’h et al. [26] and Wilkinson and Mautner [29], in Supplementary File 4.

### Intra-observer error

Intra-observer error was calculated by remeasuring CT images of 25 subjects from the n_1_ sample with a mean of 19 days intervening between measurement sessions (min = 12 days; max = 26 days). The technical error of measurement (TEM) and relative TEM (rTEM) [45–47] served as the basis for the test-retest analysis using the following equations:

2 $$\mathrm{TEM}=\sqrt{\frac{\sum_{\mathrm i=1}^{\mathrm n}\left({\mathrm x}_{1\mathrm i}-{\mathrm x}_{2\mathrm i}\right)^2}{2\mathrm n}}$$

3 $$\mathrm{rTEM}=\frac{\mathrm{TEM}}{\mathrm{mean\,of\,measurement}}\times100\%$$

Where:

*x*_*1i*_ *=* the first measurement of an individual *“i”*.

*x*_*2i*_ *=* the second repeat measurement of an individual *“i*”.

n = the sample size.

## Results

### Intra-observer error

Repeated measurements of the same scans showed TEMs to rarely exceeded 1 mm for all 19 linear measurements (Supplementary File 5). Relative TEMs were generally below 5%, with the least amount of reliability shown for the Wilkinson and Mautner [29] globe projection measurement using reference tangent between mso-mio (Supplementary File 5).

### Validation tests of previously reported estimation models (n_1_ = 58 subjects)

#### Mean linear distance models of Stephan et al. [14] and Stephan [22]

Positioning the pupil center 16.9 mm inferior to the superior orbital margin [14] produced a SEE of 2.6 mm. Positioning the pupil center 15.5 mm medial to the lateral orbital wall [14] produced a SEE of 2.3 mm. The SEE for globe protrusion, using a 16.2 mm mean distance from the dLOM [22], was 2.9 mm.

The orbit width and height measurements used in this study indicate that the oculus anterius was positioned 2.5 and 5.6 mm closer to the superior (measurement #6 − #5) and lateral orbital rims (measurement #15 − #16), respectively. This translates to oculus anterius being 1.3 and 2.8 mm more superior and lateral than the centre of the aditus orbitalis. These values are in agreement with, and next to identical to, those previously reported by Stephan et al. [14] (2.5 and 5.4 mm, respectively) and were similar laterally but smaller superiorly, than those observed by Guyomarc’h et al. [26] (2.2 and 3.0 mm, respectively).

#### Percent orbit size models of Guyomarc’h et al. [26]

Setting the ocular anterius 56.4% lateral to the medial orbital margin, 44.1% under the superior orbital rim, and projecting 51.3% of the orbit height dimension in front of the lateral orbital rim as recommended by Guyomarc’h et al. [26], resulted in the following SEEs: 1.6, 1.4 and 3.2 mm, respectively. This compares to SEE values of 1.3, 1.4 and 2.3 mm reported in Guyomarc’h et al.’s [26] paper for the Guyomarc’h et al. sample, respectively. Notable here is that SEEs for the first two measurements cross-validate Guyomarc’h et al.’s [26] findings, however, the SEE on the third measurement was not as low as that reported by the original authors.

#### Regression model of Wilkinson and Mautner [29]

When applied to this study’s sample, the regression equation of Wilkinson and Mautner [29] (globe protrusion = 18.3–0.4*orbital depth) produced a mean standard error of the estimate of 5.0 mm. This was the largest estimation error observed out of all previously published eye projection estimation models that were examined in this study.

**Fig. 4:**
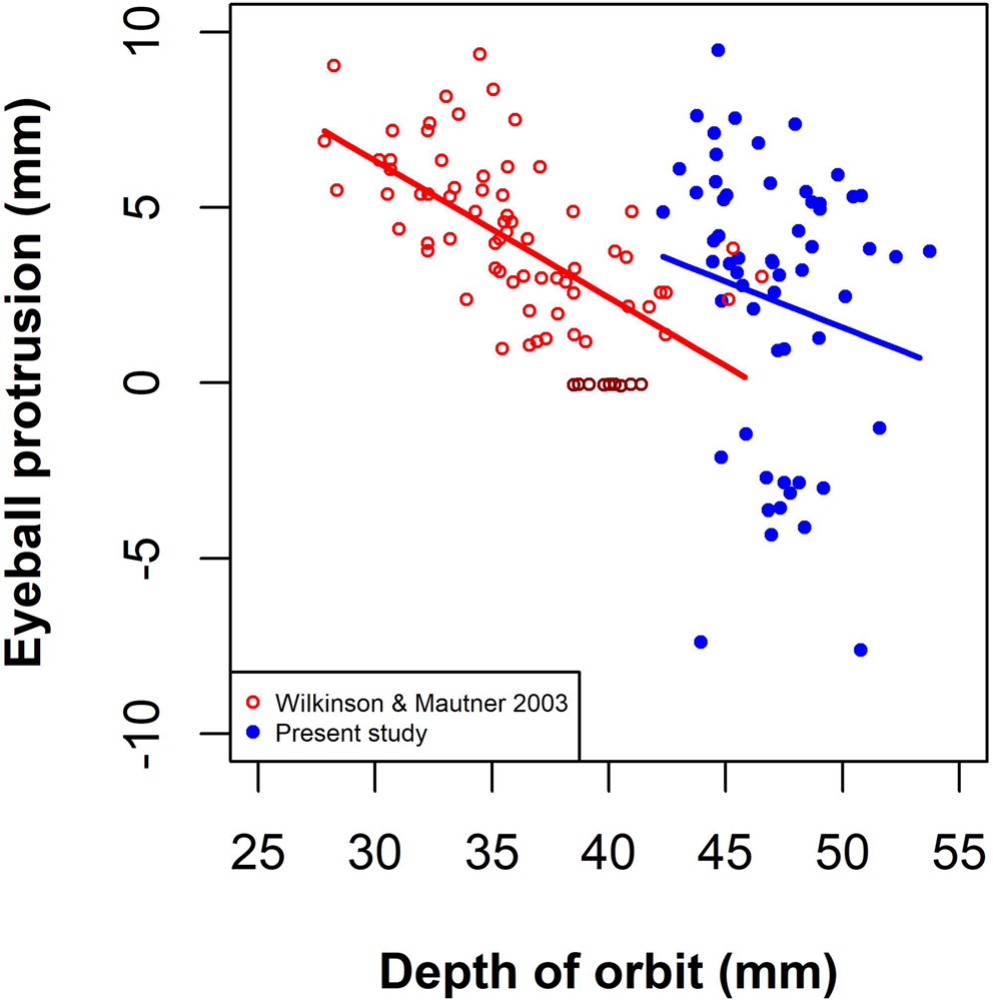
A scatterplot of this study’s orbit depth and protrusion data (closed blue circles), as measured from CTs, but otherwise in accordance with Wilkinson and Mautner’s previously published protocols [29]. These data are plotted against the artistic impression of the Wilkinson and Mautner [29] scatterplot, Fig. 2c (open red circles), for comparison. We acknowledge that this comparison is limited because the artistic impression does not represent actual or real data values of [29], only the artistic impressions assisted by plotdigitizer.com. Correlation coefficient for the red trend line is −0.64 and similar to Wilkinson and Mautner’s originally reported value of −0.646 [29]. Correlation coefficient for the blue trend line (this study) is −0.08

Correlation analysis of our sample’s data revealed a Pearson’s Product Moment Correlation Coefficient (r) of only −0.08 in contrast to Wilkinson and Mautner’s [29] originally reported −0.64 (Fig. 4) as measured with respect to the mso-mio reference plane, but as mentioned above in the methods section, note that Wilkinson and Mautner dataset holds an inflated number of entries that are dependent since both left and right sides from the same subject were included in that work. In this study, the negative correlation relationship we observed was not statistically significant (*p* = 0.248).

Unlike Wilkinson and Mautner [29], who observed eyeball protrusion values from 0 to +10 mm, we observed projection values across the full range from −7.6 to +9.5 mm relative to the reference line at the mid-sagittal anterior orbit margin (Fig. 4). We also observe for this study’s data, the unexpected clustering of data into two clouds either side of the zero line. We have thoroughly cross-checked this study’s data and found that this spread is a real reflection of the sample (i.e., the measurements are accurate). Exploration of whether or not similar clusters are observed in other larger sampled cohorts is worth pursuit in future work to characterize the reliability of this finding and what, if any, anatomical reasons underpin it (or if it is not signal, but rather just data noise). Unlike the Wilkinson and Mautner [29] study, clustering of the data on the zero line and only in the positive range above zero was not observed.

### Orbit volume relationships

#### CT segmented orbit volume

**Fig. 5:**
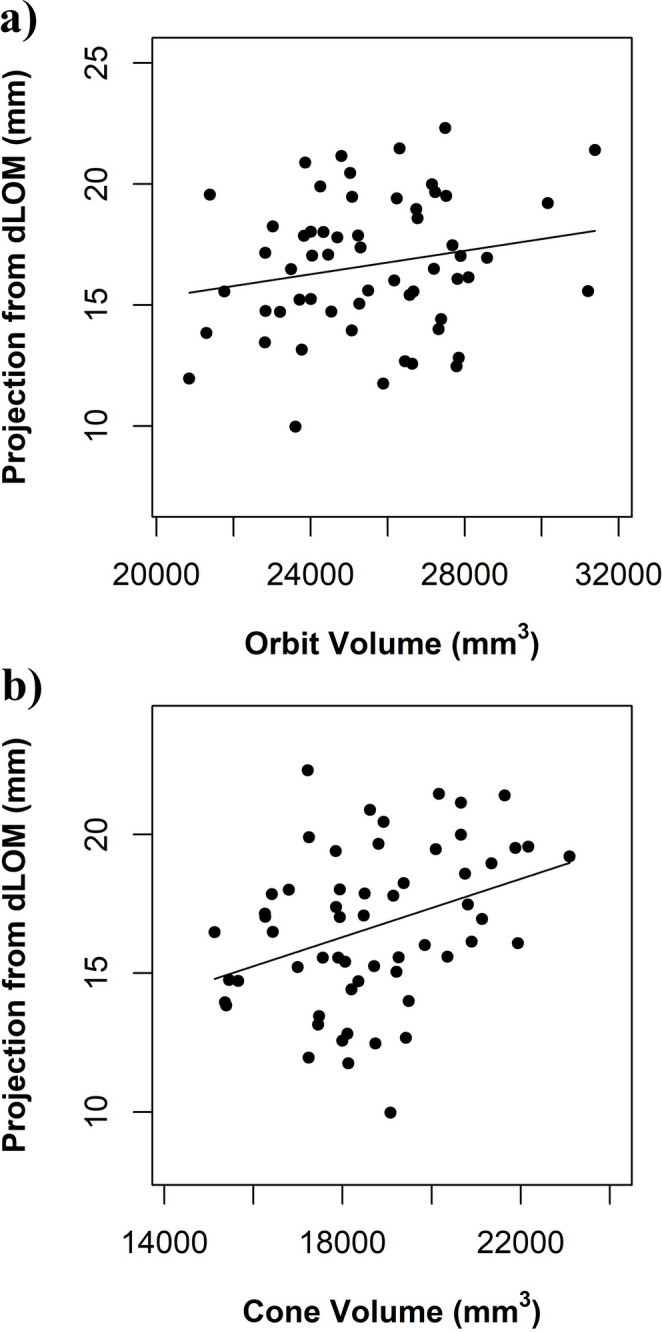
Orbit volume correlation with globe protrusion (oa-dLOM) and linear regressions: (**a**) globe protrusion with CT segmented orbit volume; and (**b**) globe protrusion with 3D cone volume as calculated from sk–or (orbit height), d–dLOM (orbit width) and sk–lOF (orbit length). The regression equation for (**a**) is: eyeball projection (dLOM-oa) = 2.43 × 10^− 4^ × Volume + 10.4 (r^2^ = 0.04, SEE = 2.8 mm). The regression equation for (**b**) is: eyeball projection (dLOM-oa) = 5.24 × 10^− 4^ × Elliptical Cone Volume + 6.85 (r^2^ = 0.13, SEE = 2.7 mm)

Segmented orbit volumes ranged from a minimum of 20,848 mm^3^ to a maximum of 31,385 mm^3^ (n_1_ = 58). The mean orbit volume was 25,555 mm^3^ with a standard deviation of 2,297 mm^3^. The anteroposterior projection from dLOM ranged from a minimum of 10.0 mm to a maximum of 22.3 mm, with a mean value of 16.6 mm and a standard deviation of 2.8 mm (n_1_ = 58). A weak, positive correlation was noted between these variables, but it was not statistically significant (*r* = 0.20, *p* = 0.14, Fig. 5).

Linear regression on the n_1_ training points produced the following estimation model for eyeball projection, as measured from dLOM: projection = 2.43 × 10^− 4^ × V + 10.4, r^2^ = 0.04, SEE = 2.8 mm, where V = segmented orbital volume (mm^3^). Out of group testing of this model using 18 additional new subjects (n_2_) cross-validated the SEE with a value of 2.1 mm.

#### Proxy orbit volume

Elliptical cone volume, calculated from the orbit height [a = sk–or], width [b = d–dLOM] and orbit length [c = sk– lOF] distance, also correlated with eyeball protrusion (*r* = 0.35, *p* < 0.01, Fig. 5) and yielded the following eyeball projection estimation model, as measured from dLOM:

projection = 5.24 × 10^− 4^ × S + 6.85, r^2^ = 0.13, SEE = 2.7 mm, where S = elliptical cone volume calculated according to Equation #1. Out-of-group testing with n_2_ produced a SEE of 2.4 mm for this model, cross-validating the training group result.

## Discussion & conclusions

This study provides a CT test of multiple estimation models for eyeball position reported across the craniofacial identification literature, whereby all models can be equivalently compared *within the same CT test sample*. This is a major advantage for benchmarking the estimation models against one another and represents the first such evaluation of its kind in the craniofacial identification domain. The study provides evidence that between 1.5 and 3.0 mm of estimation error can be expected along each of the three coordinate axes in about 68% of cases when using the highest performing estimation models for eyeball position with the least error (of course, larger error can be expected for the remaining 32% of additional cases). Poorer performing estimation models will hold higher errors.

In terms of positioning in the frontal plane, Guyomarc’h et al. [26] percentage based method for x-position (mediolateral position in the orbit) worked well, producing SEE with one millimeter less error than the next closest method. For vertical position in the orbit (z-axis position), both Guyomarc’h et al. [26] and Stephan et al. [14] methods performed equivalently. Regarding eyeball protrusion (y-axis position), grand mean exophthalmic data presented by Stephan [22] out-performed all other previously published methods, with a SEE of 2.9 mm. The regression based eyeball protrusion estimation method of Wilkinson and Mautner [29] (globe protrusion = 18.3–0.4*orbital depth) produced close to double the SEE of the mean exophthalmic data result (5 mm). As estimation models with the lowest SEE magnitudes should be awarded the highest preference for use in forensic casework, per the results of this study, the eyeball protrusion model that should be considered to be the default for craniofacial identification casework is: oculus anterius placed 16.2 mm anterior to the dLOM (SEE = 2.9 mm) as formulated from a large sample of living subjects (*n* = 1,174) [22]. We postulate that the large non-replication result observed for the Wilkinson and Mautner regression [29] may rest with regression fitting to training data that are not representative in the first instance and/or regression overfitting, which is a risk when using small samples (see Introduction and Fig. 2 for further details/discussion).

Correlation analysis of orbit volumes with globe protrusion confirms that a weak relationship exists, but it is in the opposite direction to that anticipated by Gerasimov [30, 31]. Rather than globe projection decreasing with larger orbit sizes of the closed-type and increasing with smaller shallower orbits of the open-type, the reverse is true (Fig. 5). Eyeball protrusion increases as orbit volume enlarges and for smaller orbits with smaller volume, protrusion is less. The strength of the relationship is weak, but enough that regression-based estimation models produce slightly better SEE than mean exophthalmic data. The additional trouble involved in acquiring volumetric data from CT scans for entry to the regression equations is unlikely to justify the marginal improvements, or override the practical utility offered by a simple exophthalmic mean which performs with similar accuracy. For this reason, we do not recommend the casework employment of the additional regression-based models for estimating globe projection that were derived in this study from the CT obtained orbit volumes.

The 3D elliptical cone approximation that used only three input linear measurements circumvented much of the trouble of CT volumetric measurement, but the SEE improvements were small for the additional complexity/inconvenience in contrast to the much simpler (and almost equally accurate) grand mean exophthalmos acquired from living subjects. The fact that volumetric relationships with globe projection are in the opposite direction to those anticipated by Gerasimov, indicates that Gerasimov’s formulations for this parameter, should receive less attention moving forwards. The globe protrusion relationship with volumetric orbit size is in a weak positive direction.

As scientific confidence in estimation models is built through validation testing using independent samples [48], repetition of this study using additional samples (including younger living subjects studied by CBCT and or MRI) would be valuable. In terms of volumetric analyses, these studies should ideally engage similar high-resolution scans and thin slice thicknesses to retain representativeness to this study, however it should be noted that minor differences in segmentation protocols (e.g., inclusion or exclusion of nasolacrimal ducts) or scan thicknesses are unlikely to interfere with the direction of the results overall so long as segmentation technique is consistent within each study. In particular, MRI studies of living subjects would add value, however reduced image clarity may be a complicating factor as it acts to increase measurement uncertainty. High-resolution MRI head scans are not a fast undertaking (60+ seconds), such that movements during breathing, blood pulse, blinking, heartbeat can blur fine details of the globe as mentioned in the Materials and Methods section. Discounting noisy images is one strategy to reduce impacts, however, this reduces sample size overall, meaning larger starting samples of living subjects are ideally required for equivalent statistical sensitivity. Investigating relationships with CBCT in upright living subjects would be another useful pursuit, however, this method too incorporates limitations that ultimately forces compromises. For example, image clarity/resolution of CBCT is typically less than that of thin slice regular CT and head stabilization attachments such as chin cup rests or bite plates in CBCT may apply unwanted forces to the facial soft tissues and modify the soft tissue drape, including that at the eyes. These variables will need to be contended with in future studies.

While within-group test data that are used to train estimation models provide some sense of accuracy when used independently, this study underscores why accuracy results from training data alone should not be used to allocate accuracy or reliability exclusively. Training data tend to provide inflated accuracies of their associated models because the model was specifically formulated for (derived on) that exact dataset. Recent blanket recommendations for eyeball position models based on percent orbit size [49] and only on findings from original training set data [26] are a case in point. This study clearly shows that the percentage-based models do not always produce the smallest estimation errors (e.g., for globe protrusion and some horizontal distances) and other estimators quantitatively perform equally well or better per cross-validation results using out-of-group samples. Only one of three percent-based models for globe position out-performed other simpler means-based models in this study, and in one case (globe projection) the percent model performed substantially worse.

In this CT study, attention concerned adults and the sample was skewed towards those of older age (65+ years). This study thereby provides critical tests of the generalizability of established estimation models to this cohort specifically. The door is open, however, for further cross-validation testing to further elucidate the extent of generalizability of models across the full scope of the adult context, including individuals as young as 18 years. As outlined above, these tests using younger living adults carry some challenges, but their results will irrespectively value add to the larger craniofacial identification picture. Of course, the most ideal estimation models will be those that are broadly generalizable to all adults with little error, such that they can be used in a multitude of contexts with confidence. It is notable that in this study we observed some estimation models, which were derived on young adult samples, to hold good generalizability to the older adult sample (e.g., 16.2 mm mean distance from the dLOM for eyeball protrusion [22]), however, this was not the case for all estimation models (e.g., globe protrusion = 18.3–0.4*orbital depth after [29]).

It is clear that special care should be taken to avoid the presentation of incompletely tested estimation models prematurely as formalized craniofacial identification ‘standards’. The label of ‘standard’ must be reserved for only those methods that have stood up to comprehensive scrutiny and are accompanied by quantitative data (not practitioner whims) that justify their reliability and accuracy claim. The near complete absence of out-of-group test data for many estimation models in the craniofacial identification domain, despite the reporting of so-called standards [49, 50] (including that for the eyeball), suggests that the status of the discipline is much more fluid than well-agreed and/or established rules. Rather, justified preferences for models are likely to only be established once sizeable cross-validation data emerge, ideally from multiple measurement modalities and samples, with the result that the standard errors settle towards a consistent value as they become more reliable. These studies take time and will likely be conducted in series. In this context, standard errors should not be regarded, at this stage, to represent fixed numbers. Rather, standard errors are likely to ebb, flow and be modified as new additional validation studies are conducted, data emerge and overarching assessments are updated. Only with cross-validation testing will models worthy of a label signifying a “standard” status (based on the data criteria of low standard errors) be warranted. It is useful to note that in deriving estimation models in the first instance, representative samples of large size are generally preferred, such that the risk of model over-fitting is reduced [51, 52]. In terms of validation testing, this study has made a start for eyeball estimators and tests of rules for other facial features must follow in future work.

## Supplementary Information

Below is the link to the electronic supplementary material.

Supplementary Material 1

Supplementary Material 2

Supplementary Material 3

Supplementary Material 4

Supplementary Material 5
