# Supplementary material for: A computed tomography (CT) study of the eyeball position and estimation models for craniofacial identification
Source: Int J Legal Med. 2026 Apr 10;140(4):2395–406. doi: 10.1007/s00414-026-03768-3 (PMC13275775; doi:10.1007/s00414-026-03768-3)
Supplement: Supplementary file 1 — Supplementary Material 1 [file 414_2026_3768_MOESM1_ESM.pdf]

Supplementary File 1: Definitions of the 11 orbital landmarks used in this study.

Landmark Name	Abbreviation	Definition
Mid-supraorbital	mso [1]	The most anterior point on the superior orbital rim set midway and equidistantly between dacryon and dLOM*
Mid-infraorbital	mio [1]	The most anterior point on the inferior orbital rim set midway and equidistantly between dacryon and dLOM*
Nasion	n [2]	Intersection of the nasofrontal sutures in the median plane
Dacryon	d [2]	The point of juncture between sutures of the frontal, maxillary and lacrimal bones
Flower's point	FP [3]	The point of intersection between the posterior lacrimal crest and the frontal bone
Supraconchion	sk [4]	Point at the maximum of margo supraorbitalis, excluding a supraorbital notch if present, and on the most inferior aspect of the ridge anteroposteriorly*
Lateral aspect of the Lateral Orbital Margin	lLOM [5]	Most lateral portion of the lateral orbital margin, placed at the anterior apex of the rim's curve
Ectoconchion	ec [4]	The point on the lateral orbital margin following a line that obliquely bisects the orbit from dacryon
Deepest point of the Lateral Orbital Margin	dLOM [5]	Deepest portion of the lateral orbital margin, placed at the anterior apex of the rim's curve
Orbitale	Or [2]	Point at the minimum of margo infraorbitalis, on the most superior aspect of the ridge anteroposteriorly
Lateral aspect of Optic Foramen	lOF*	The most lateral aspect at the most superficial portion of the optic foramen*

* Landmark and/or definition represents the authors' own formulation for this study.

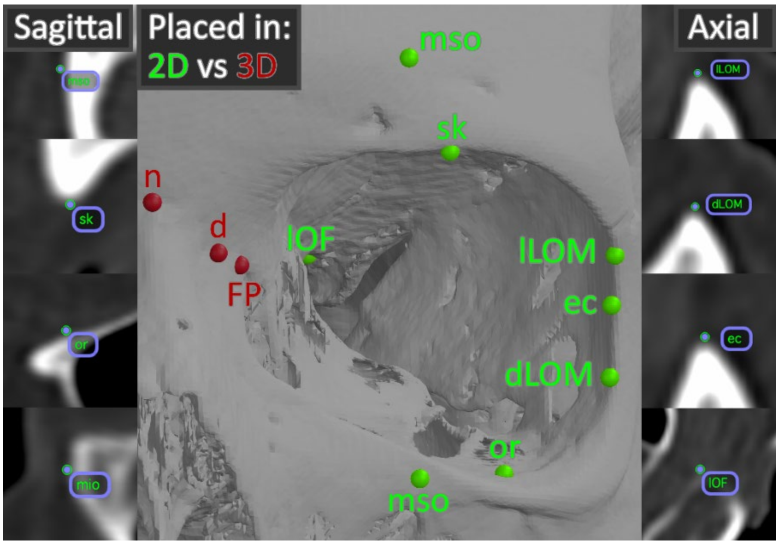

Figure 1: Illustration of the 11 orbital landmarks defined above. Main central image shows all landmarks used as located on rendered CT. Note some landmarks are placed using 2D raw axial orthoslices and 2D sagittal plane reconstructions (green), not using the 3D render, per the thumbnail views on left and right margins.

References

1. Stephan CN, Simpson EK (2008) Facial soft tissue depths in craniofacial identification (Part I): An analytical review of the published adult data. J Forensic Sci 53: 1257-72. doi: https://doi.org/10.1111/j.1556-4029.2008.00852.x
2. Martin R. (1928) Lehrbuch der Anthroplogie. Gustav Fischer Jena.
3. Whitnall SE. (1932) The Anatomy of the Human Orbit and Accessory Organs of Vision. 2nd ed. Oxford University Press London.
4. Guyomarc'h P, Dutailly B, Couture C, Coqueugniot H (2012) Anatomical placement of the human eyeball in the orbit-validation using CT scans of living adults and prediction for facial approximation. J Forensic Sci 57: 1271-5. doi: https://doi.org/10.1111/j.1556-4029.2012.02075.x
5. Stephan CN, Davidson PL (2008) The placement of the human eyeball and canthi in craniofacial identification. J Forensic Sci 53: 612-9. doi: https://doi.org/10.1111/j.1556-4029.2008.00718.x
