# Supplementary material for: A computed tomography (CT) study of the eyeball position and estimation models for craniofacial identification
Source: Int J Legal Med. 2026 Apr 10;140(4):2395–406. doi: 10.1007/s00414-026-03768-3 (PMC13275775; doi:10.1007/s00414-026-03768-3)
Supplement: Supplementary file 2 — Supplementary Material 2 [file 414_2026_3768_MOESM2_ESM.pdf]

Supplementary File 2: The 20 measurements undertaken in this study

#	Measurement	Measurement Name	Units
1	*mso-mio*	Orbital rim height	mm
2	*mso-mio* [superior]	-	mm
3	*mso-mio* [inferior]	-	mm
4	*sk-or*	Orbit height	mm
5	*sk-or* [superior]	-	mm
6	*sk-or* [inferior]	-	mm
7	*dLOM-d*	Orbit width	mm
8	*dLOM-d* [medial]	-	mm
9	*dLOM-d* [lateral]	-	mm
10	*dLOM-oa*	Eyeball projection	mm
11	*ec-d*	-	mm
12	*ec-d* [medial]	-	mm
13	*ec-d* [lateral]	-	mm
14	*FP-lLOM*	*Orbit breadth*	mm
15	*FP-lLOM* [medial]	-	mm
16	*FP-lLOM* [lateral]	-	mm
17	mso-mio orbit depth after Wilkinson and Mautner [29]	Wilkinson and Mautner [29] orbit depth	mm
18	mso-mio globe projection after Wilkinson and Mautner [29]	Wilkinson and Mautner [29] eyeball projection	mm
19	*sk-lOF*	Anteroposterior orbit length	mm
20	orbital volume	orbital volume	mm^3

Note:
Orbital rim height (#1) = #2 + #3
Orbit height (#4) = #5 + #6
Orbit width (#7) = #8 + #9
Orbit breadth (#14) = #15 + #16
2 + 3 = b in Fig. 3(c)
5 + 6 = a in Fig. 3(c)
19 = c in Fig. 3(c)

Illustrated examples of the 19 linear measurements above on multi-slice reconstructions from the CT:

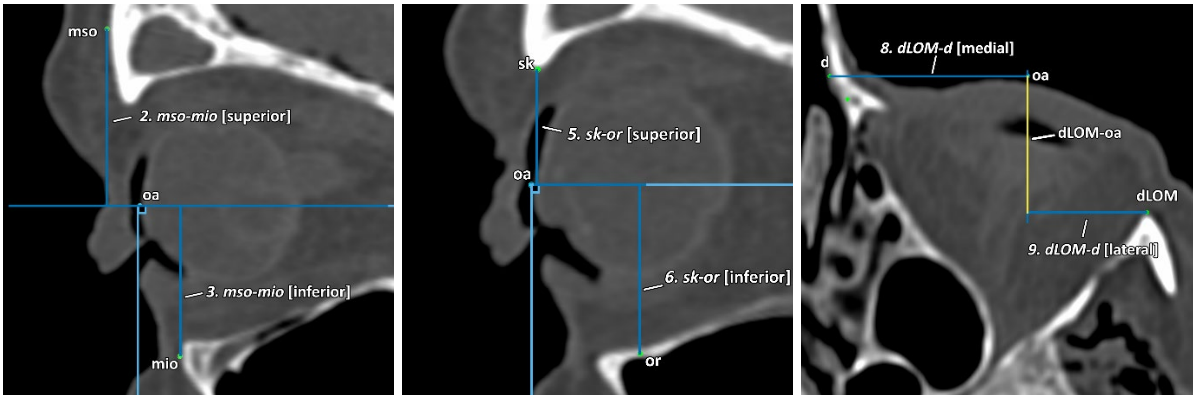

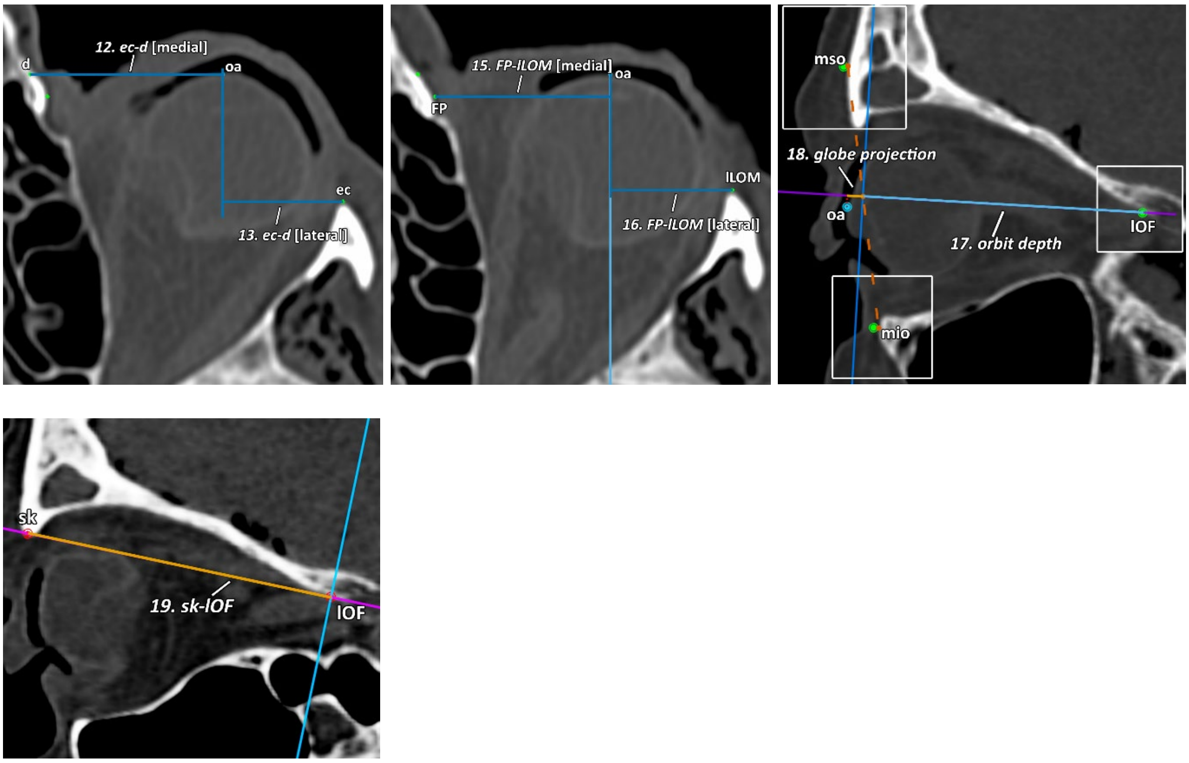
12. ec-d [medial]
d
oa
ec
13. ec-d [lateral]
15. FP-ILOM [medial]
oa
FP
ILOM
16. FP-ILOM [lateral]
mso
18. globe projection
oa
17. orbit depth
IOF
mio
sk
19. sk-IOF
IOF
