# Supplementary material for: A computed tomography (CT) study of the eyeball position and estimation models for craniofacial identification
Source: Int J Legal Med. 2026 Apr 10;140(4):2395–406. doi: 10.1007/s00414-026-03768-3 (PMC13275775; doi:10.1007/s00414-026-03768-3)
Supplement: Supplementary file 4 — Supplementary Material 4 [file 414_2026_3768_MOESM4_ESM.pdf]

Supplementary File 4: CT Ground Truth Mean (CT_GT), mean of the estimate values ($Mean_{est}$) and standard error of the estimate (SEE) for measurements representing the estimation models across all samples examined in this study.

#	Author Method	Measurement	n_1 (n=58)			n_2 (n=18)			n_3 (n=76)		
			CT_GT (mm)	$Mean_{est}$ (mm)	SEE (mm)	CT_GT (mm)	$Mean_{est}$ (mm)	SEE (mm)	CT_GT (mm)	$Mean_{est}$ (mm)	SEE (mm)
1	**Guyomarc'h et al. [26]**	Mediolateral % (d-oa)	24.9	23.7	1.6	24.7	23.4	1.9	24.9	23.7	1.6
2		Superoinferior % (sk-oa)	16.5	15.7	1.4	16.6	15.6	1.4	16.5	15.6	1.4
3		Anteroposterior % (dLOM-oa)	16.6	18.2	3.2	17.3	18.1	2.3	16.8	18.2	3.0
4	**Stephan et al. [14]**	FP-oa (ML)	22.9	20.9	2.6	23.1	20.9	2.9	22.9	20.9	2.6
5		lLOM-oa (ML)	17.3	15.5	2.3	16.6	15.5	1.9	17.1	15.5	2.2
6		sk-oa (SI)	16.5	16.9	1.7	16.6	16.9	1.7	16.5	16.9	1.7
7		or-oa (SI)	19.0	19.4	1.5	18.8	19.4	1.5	19.0	19.4	1.5
8	**Stephan [22]**	dLOM-oa (AP projection)	16.6	15.9	2.9	17.3	15.9	2.6	16.8	15.9	2.8
9	**Wilkinson & Mautner [29]**	Wilkinson & Mautner projection	2.6	-0.53	5.0	4.1	-0.1	4.8	2.91	-0.43	4.9
10	**This Study**	AP projection from segmented orbit volume	16.6	16.6	2.8	17.3	16.6	2.1	16.8	16.6	2.6
11		AP projection from cone volume estimates	16.6	16.6	2.7	17.3	16.3	2.4	16.8	16.6	2.6
