# Supplementary material for: A computed tomography (CT) study of the eyeball position and estimation models for craniofacial identification
Source: Int J Legal Med. 2026 Apr 10;140(4):2395–406. doi: 10.1007/s00414-026-03768-3 (PMC13275775; doi:10.1007/s00414-026-03768-3)
Supplement: Supplementary file 5 — Supplementary Material 5 [file 414_2026_3768_MOESM5_ESM.pdf]

Supplementary File 5: Summary statistics of measurements and associated technical errors of measurement (TEM).

						Intra-observer Error	
	Measurement	x̄ (mm)	*s* (mm)	Min (mm)	Max (mm)	TEM (mm)	rTEM (%)
1	*mso-mio*	45.5	3.7	36.7	54.5	1.0	2
2	*mso-mio oa* [superior]	24.8	3.4	16.7	32.2	1.1	4
3	*mso-mio oa* [inferior]	20.7	2.2	16.3	25.1	0.6	3
4	*sk-or*	35.5	2.2	30.8	40.2	0.6	2
5	*sk-or oa* [superior]	16.5	1.7	11.9	20.1	0.6	4
6	*sk-or oa* [inferior]	19.0	1.5	14.2	22.2	0.4	2
7	*dLOM-d*	41.2	2.0	37.1	45.4	0.6	2
8	*dLOM-d oa* [medial]	24.9	1.8	21.2	28.9	0.7	3
9	*dLOM-d oa* [lateral]	16.3	1.3	13.1	19.3	0.3	2
10	*dLOM-oa*	16.6	2.8	10.0	22.3	0.5	3
11	*ec-d*	41.4	1.9	37.5	46.2	0.6	1
12	*ec-d oa* [medial]	24.9	1.8	21.2	28.9	0.6	2
13	*ec-d oa* [lateral]	16.5	1.3	13.7	19.6	0.3	2
14	*FP-lLOM*	40.2	1.6	36.9	43.5	0.4	1
15	*FP-oa* [medial]	22.9	1.6	18.5	26.2	0.5	2
16	*lLOM-oa* [lateral]	17.3	1.5	14.5	22.2	0.3	2
17	mso-mio orbit depth [29]	47.1	2.5	42.3	53.7	0.5	1
18	mso-mio globe projection [29]	2.6	4.0	-7.6	9.5	0.4	13
19	sk-lOF	48.7	2.6	43.7	56.1	0.8	2
20	orbital volume	25,555	2,297	20,848	31,385	617	2
